# Next-generation RNA sequencing elucidates transcriptomic signatures of pathophysiologic nerve regeneration

**DOI:** 10.1038/s41598-023-35606-6

**Published:** 2023-05-31

**Authors:** Wesley S. Warner, Christopher Stubben, Stewart Yeoh, Alan R. Light, Mark A. Mahan

**Affiliations:** 1 Department of Neurosurgery, Clinical Neurosciences Center, The University of Utah, 175 North Medical Dr. East, Salt Lake City, UT 84132 USA; 2 Bioinformatics Shared Resource, Huntsman Cancer Institute, University of Utah, Salt Lake City, USA; 3 Department of Anesthesiology, University of Utah, Salt Lake City, UT USA

**Keywords:** Peripheral nervous system, Next-generation sequencing, RNA sequencing, Neuroimmunology, Trauma, Regeneration and repair in the nervous system

## Abstract

The cellular and molecular underpinnings of Wallerian degeneration have been robustly explored in laboratory models of successful nerve regeneration. In contrast, there is limited interrogation of failed regeneration, which is the challenge facing clinical practice. Specifically, we lack insight on the pathophysiologic mechanisms that lead to the formation of neuromas-in-continuity (NIC). To address this knowledge gap, we have developed and validated a novel basic science model of rapid-stretch nerve injury, which provides a biofidelic injury with NIC development and incomplete neurologic recovery. In this study, we applied next-generation RNA sequencing to elucidate the temporal transcriptional landscape of pathophysiologic nerve regeneration. To corroborate genetic analysis, nerves were subject to immunofluorescent staining for transcripts representative of the prominent biological pathways identified. Pathophysiologic nerve regeneration produces substantially altered genetic profiles both temporally and in the mature neuroma microenvironment, in contrast to the coordinated genetic signatures of Wallerian degeneration and successful regeneration. To our knowledge, this study presents as the first transcriptional study of NIC pathophysiology and has identified cellular death, fibrosis, neurodegeneration, metabolism, and unresolved inflammatory signatures that diverge from pathways elaborated by traditional models of successful nerve regeneration.

## Introduction

Traumatic peripheral nerve injury remains a challenging clinical dilemma because of the frequent failure of nerve regeneration, and consequently, limited neurologic recovery^1,2^. Despite the innate genetic regeneration programs of peripheral nerves^3,4^, severe traumatic injuries often result in the formation of a neuroma or neuroma-in-continuity (NIC), a pathophysiologic consequence hallmarked by perineurial encapsulation of small unmyelinated axons, failed distal axon regeneration, robust fibrotic tissue deposition, and aberrant ion channel distribution^5–8^. Persistent presence of immune cells, such as phagocytic macrophages and T-cells, has also been observed in the context of human neuroma specimens and is implicated in potentiating painful phenotypes^9–11^. This microenvironment is believed to inhibit axonal growth and may be analogous to the pathophysiology of spinal cord injury, where axonal regeneration is limited by scar formation, unresolved inflammation, and a dearth of trophic support molecules^12,13^. Unfortunately, beyond the categorization of neuroma pathology, little is understood about the mechanisms precipitating neuroma formation and associated regenerative failure.

Advances in next-generation RNA sequencing (RNAseq) have elucidated pivotal insights underpinning homeostasis and pathogenesis while identifying specific targets for mechanistic perturbation and therapeutic development^14^. Application of these tools has recently shifted to the processes of neural regeneration, with examination of the molecular portraits of brain^15–17^, spinal cord^18–20^, and dorsal root ganglia^21–23^ to enhance our understanding of the activated transcriptional programs associated with successful nerve regeneration. Studies have also elucidated the molecular programs governing regeneration in peripheral nerves, specifically highlighting functional and biological pathways in Wallerian degeneration (WD) that lead to axon regeneration and functional recovery^24–29^. Lamentably, these insights in successful nerve regeneration models may not be applicable to the dismal failures of clinical injuries, as evidenced by the dearth of therapeutic intervention strategies^30^. Therefore, deconstructing the molecular underpinnings of the pathophysiologic condition may reveal novel transcriptional signatures to guide prognostic biomarker discovery or identify mechanistic targets to prevent neuroma formation and imminent neurologic consequences, such as chronic pain and limited functional recovery.

To address the translational gap from basic science models to clinical injuries, we have created and validated a laboratory model of rapid-stretch nerve injury, which is the predominant mechanism associated with traumatic nerve injury and neuroma formation in the clinical setting^2^. Biomechanical^31^, histologic^32^, and behavioral^33^ outcomes faithfully recapitulate the neuroma phenotype and neurologic deficits found in the clinical population. Expanded comparison to traditional models of crush, transection, and delayed transection repair has delineated recovery profiles specific to the rapid-stretch mechanism of injury and associated neuroma pathophysiology^34^. To further this understanding at the molecular level, we sought to elucidate genetic signatures associated with NIC formation and identify critical pathways associated with the transition from favorable regeneration towards pathologic remodeling. Here, we present insights into the aberrant regenerative program through a temporal examination of genetic activity and application to downstream biological and functional pathways.

## Results

### Survey of genomic expression profiles

**Figure 1:**
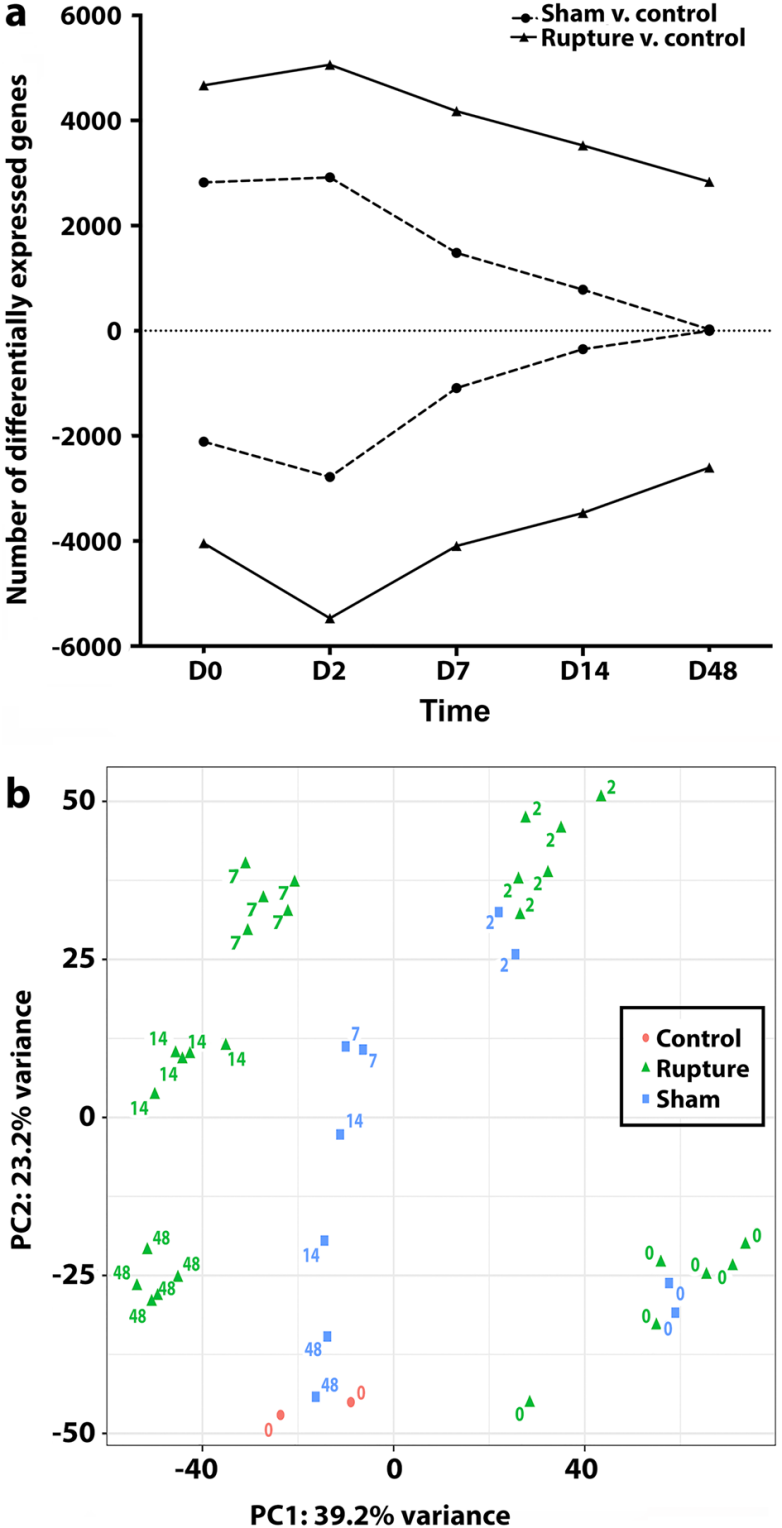
Global genetic expression profiles of differentially expressed genes across timepoints. (**a**) Differential expression is determined by DESeq analysis (DESeq2 version 1.30.0) (https://bioconductor.org/packages/release/bioc/html/DESeq2.html) with a false discovery rate of 5% and adjusted *p*-value < .05. A dramatic increase in genetic activity is observed 6 h after injury in both Rupture and Sham injury states. Day 2 (D2) demonstrates peak activity, followed by a linear decline for both comparisons. For Rupture vs. Control, although there is a temporal decrease in activity, 2834 genes remain upregulated and 2603 downregulated at the terminal timepoint, 48 days after injury. In comparison, Sham returns to Control expression levels. The activity of up- and downregulated genes present antithetical activation patterns. (**b**) The PCA plot is a dimensional reduction method to visualize similarity of genetic expression profiles in the dataset (RStudio 4.2.1: highcharter 0.9.4. https://cran.r-project.org/web/packages/highcharter/index.html). Rupture and Sham demonstrate similar clustering for days 0 and 2, while divergence ensues by day 7. After this schism, rupture injury demonstrates a unique clustering pattern for the remaining timepoints. Distinct clustering profiles are observed for all timepoints, aside from the expected Sham clustering with Control by day 48, which reflects return to homeostasis.

After rupture injury, there was a dramatic increase in genetic activity, with upregulated genes peaking at day 2 (D2) and declining linearly over time; this pattern was mirrored by downregulated genetic activity patterns (Fig. 1a). Despite the decrease in differentially expressed genes (DEGs), at the final timepoint (D48) there remained a large number of DEGs versus baseline. In contrast, although sham injury also demonstrated marked increase in DEGs in the acute phase after surgery, DEGs returned to baseline expression patterns.

Global changes in genetic expression were further delineated through principal component analysis (PCA), which aims to deconstruct multidimensional differences in gene expression to elucidate similarities in genetic activity profiles (Fig. 1b). Collectively, samples for each timepoint for the different injury severities demonstrated reliable clustering patterns, except that the sham injury was indistinct from control by D48. Interestingly, sham and rupture demonstrated relatively tight clustering for D0 and D2, then decoupled into distinct profiles for the remaining timepoints, indicative of unique genetic programs starting by D7.

**Figure 2:**
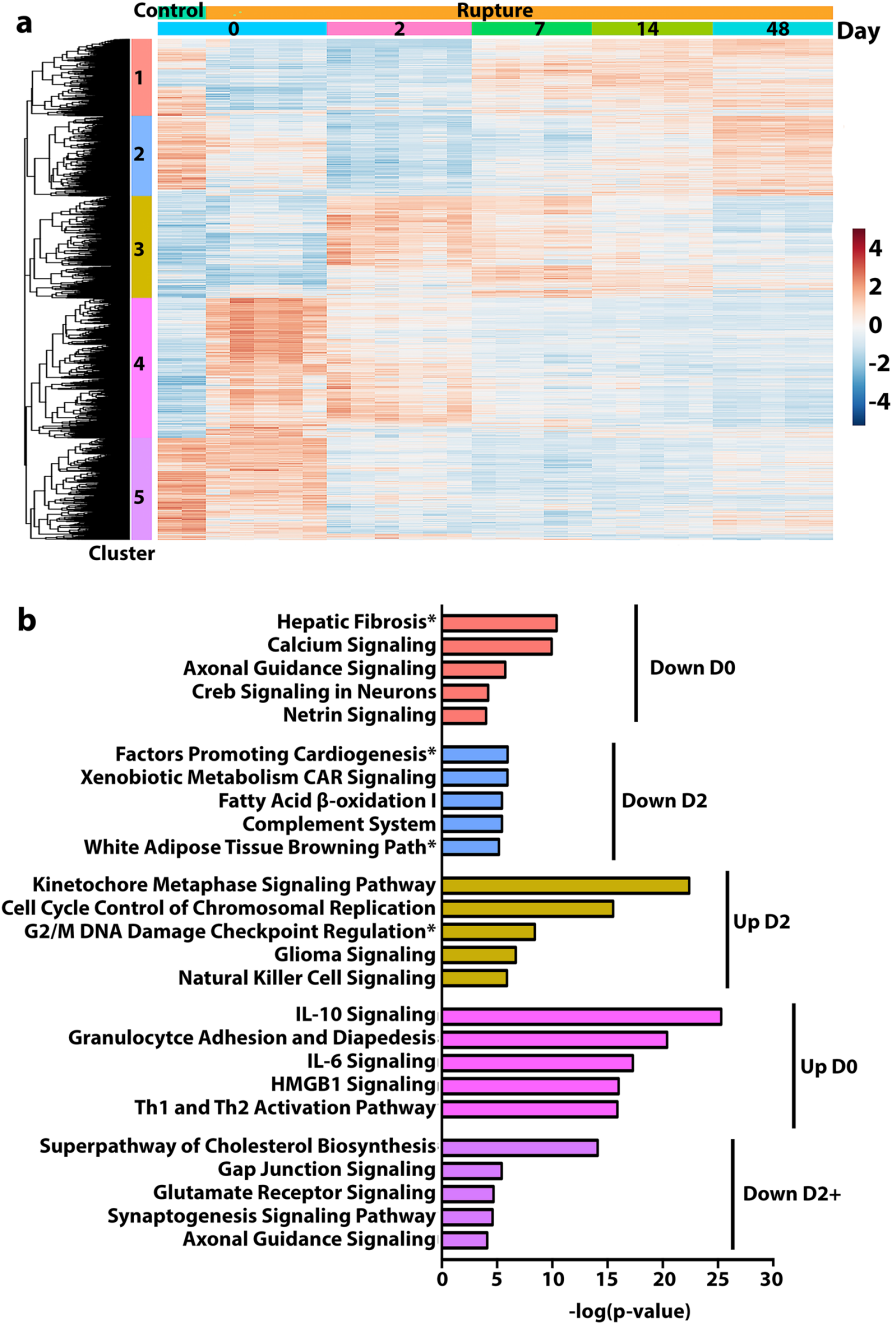
Cluster analysis to visualize the broad temporal patterns of genetic expression profiles with similar activity and application to IPA canonical pathways. (**a**) Cluster analysis and heatmap representation identified 5 clusters of genes demonstrating similar expression patterns across experimental timepoints (RStudio 4.2.1: pheatmap 1.0.12. https://cran.r-project.org/web/packages/pheatmap/index.html). Clusters 1 and 4 reflect immediate changes in activity patterns, with Cluster 1 switching from up- to downregulation by 6 h after injury and Cluster 4 demonstrating the inverse pattern of down- to upregulation. Clusters 2, 3, and 5 depict delayed changes in activity; Clusters 2 and 5 express upregulated activity for control and then transition to downregulation by D2, while the inverse is observed for Cluster 3. Uniquely, Cluster 5 remains the most distinct from control expression levels at the terminal timepoint, while the other clusters return to similar expression profiles as their control counterparts. (**b**) Genes derived from the clustering were then subjected to IPA canonical pathways analysis (QIAGEN Inc., https://digitalinsights.qiagen.com/IPA) to elucidate representative pathways, with the top 5 significant pathways presented. Down D0 (Cluster 1) genes mostly associate with fibrosis pathways and those implicated in neuronal specific activity. Metabolic regulation paths align with Down D2 (Cluster 2), whereas cell cycle regulation pathways are implicated with Up D2 (Cluster 3). Immune-related signatures dominate Up D0 (Cluster 4) whereas Down D2 + (Cluster 5) implicates pathways with both metabolic and neuronal underpinnings.

Cluster analysis through Euclidian distance matrix mapping was performed to identify gene clusters demonstrating similar temporal activity profiles (Fig. 2a). Five clusters were distinguished that broadly bifurcated into 2 temporal activation patterns: (1) immediate up- or downregulation (Clusters 1 and 4) and (2) delayed up- or downregulation (Clusters 2, 3, 5). Gene clusters were then subject to Ingenuity Pathway Analysis (IPA) to identify metabolic and canonical signature pathways (Fig. 2b).

The immediate downregulated genes (Cluster 1; decreased D0 with partial return to control expression at D48) were grossly associated with neuronal signaling pathways, such as Axonal Guidance Signaling and Netrin Signaling (Fig. 2b). Immediate upregulated genes (Cluster 4; increased D0 with partial decrease to control expression at D48) demonstrated immunologic signatures, with the top statistically significant pathways implicating cytokine signaling (IL-10, IL-6), T cell activity (Th1 and Th2 Activation Pathway), and the innate immune response (Granulocyte Adhesion and Diapedesis, HMGB1 Signaling). Delayed downregulation genes (Cluster 2; decreased D2 with partial return to control expression at D48) aligned with pathways implicated in metabolic regulation. Cluster 3 (increased D2 with partial return to control expression at D48) genes were associated with cell-cycle pathways and a second wave of the immune response. Finally, Cluster 5 (decreased D2 and no substantial return to control expression at D48) genes were related to pathways primarily involved in neuronal processes, such as Synaptogenesis and Glutamate Receptor Signaling, although the most significant path was associated with metabolic dysregulation, i.e., Superpathway of Cholesterol Biosynthesis. Importantly, in contrast to the other clusters, which chiefly resolved towards the control activity state, Cluster 5 genes had persistently decreased expression and involved axonal pathways in IPA canonical pathways analysis.

### Pathway and functional analysis of differentially expressed genes at distinct timepoints

To develop a framework for understanding pathways involved in neuroma formation, DEGs were subject to Gene Set Enrichment Analysis (GSEA), IPA canonical pathways analysis, and IPA diseases and functions analysis. Utilization of two different analysis platforms, each with unique methods of attributing genetic activity to pathways, permits a corroborative framework for results.

#### GSEA

**Figure 3:**
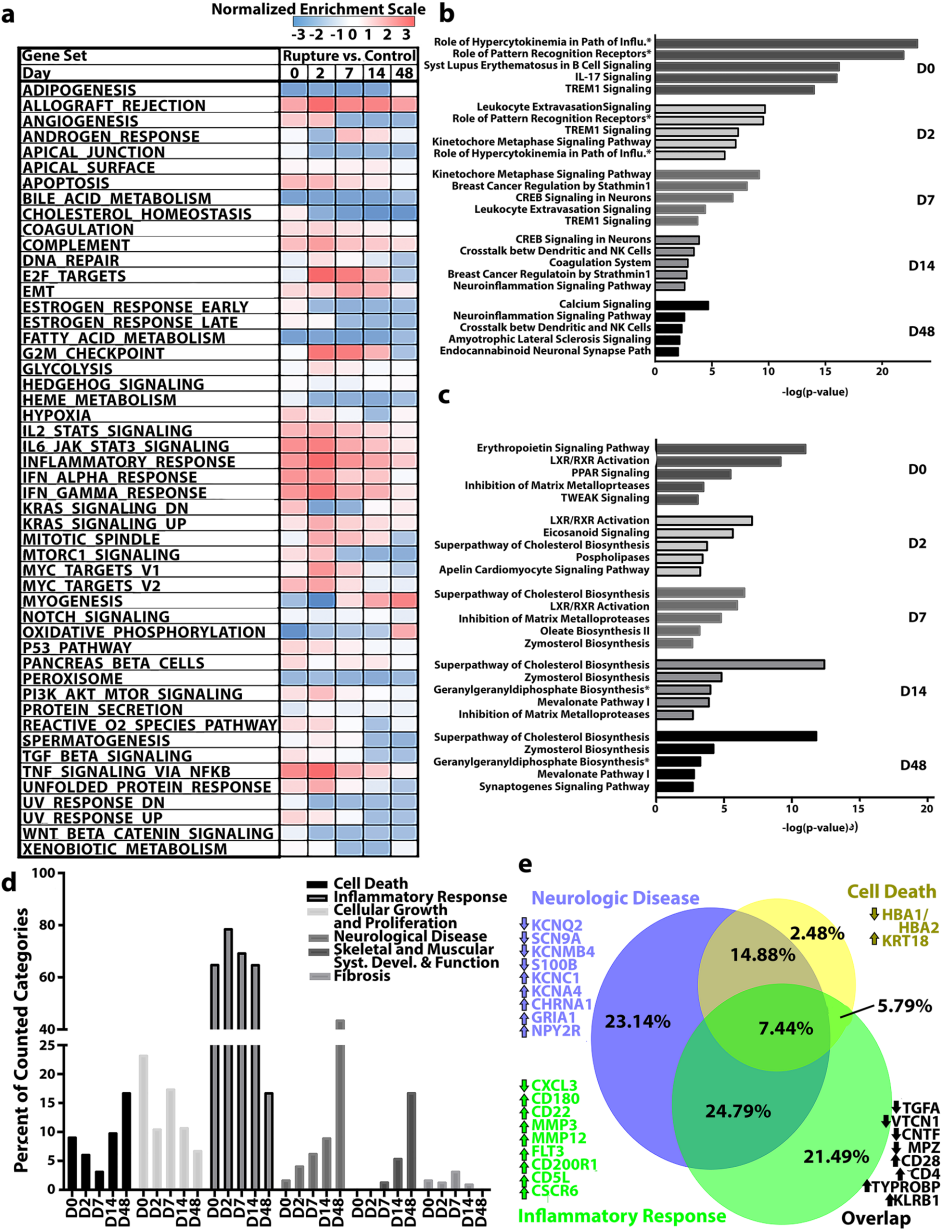
Temporal genetic signatures of pathologic neuroma-in-continuity formation subject to pathway analysis. (**a**) GSEA (version 4.1.0, Broad Institute, San Diego, CA) presents positive enrichment of pathways involved in the inflammatory response through the terminal timepoint of day 48 (D48). Pathways associated with negative enrichment are profusely metabolic. (**b**) Top 5 statistically significant (*p* < .05) IPA canonical pathways (QIAGEN Inc., https://digitalinsights.qiagen.com/IPA) upregulated at each timepoint. For D0 and D2, a majority of pathways are associated with inflammatory response. Cell cycle and neuronal pathways emerge at D7 and persist through D14. At the terminal timepoint (D48) and neuroma pathology, DEGs align with neurodegenerative pathways while the inflammatory response remains enriched. (**c**) Top 5 statistically significant IPA canonical pathways downregulated at each timepoint, which are predominately metabolic. (**d**) Through IPA diseases and functions analysis, we identified six categories associated with neuroma formation across timepoints. Sub-annotations of specific diseases and functions, out of total number of sub-annotations per day, were used to create a percent representation for each category. Inflammatory response is highly implicated for all timepoints. Both the muscle and neurologic disease categories increase in percent representation overtime. Fibrosis was limited in presentation compared with other categories, yet present for timepoints D0 through D14. Finally, both cell death and cellular growth and proliferation were depicted across all timepoints. (**e**) At the terminal endpoint, the neuroma microenvironment is highly associated with categories involved in cell death, neurologic disease, and the inflammatory response. Representative genes associated with the top functions are indicated.

DEGs between the rupture and control groups were subjected to GSEA analysis, broadly demonstrating early and prolonged upregulation of inflammatory gene sets such as Complement, IFNγ Response, and TNFα Signaling via NFκβ in the rupture group. A modest increase in angiogenesis occurred at early timepoints, while a notable increase in genes associated with myogenesis and oxidative phosphorylation occurred from D7 to D48. Consistent upregulation of Apoptosis and Epithelial-Mesenchymal Transition was also present. In contrast, pathways associated with metabolism were downregulated across all experimental timepoints (Fig. 3a).

#### Ingenuity canonical pathways analysis of DEGs

IPA canonical pathway analysis applies the experimentally derived genetic expression profiles to well-defined canonical cell signaling and metabolic pathways to identify significant associations and predicted activation states (Fig. 3b,c). In the acute phase after injury (D0 and D2), the majority of upregulated canonical pathways were significantly associated with the inflammatory response, such as TREM1 Signaling and IL-17 Signaling. In comparison, D7 was hallmarked by increased paths associated with the cell cycle and paths associated with neuronal activity, such as CREB Signaling in Neurons. Integration of neuronal, inflammatory, and cell-cycle pathways presented for significant upregulation for D14. Finally, persistent inflammatory response on D48 was highlighted by significant upregulation of pathways such as Neuroinflammation Signaling (Fig. 3b). Downregulated pathways were predominately associated with metabolism (Fig. 3c). Inhibition of extracellular matrix degradation was also implicated, with significant downregulation of the Inhibition of Matrix Metalloproteases for multiple timepoints (D0, D7, D14), while axonal regeneration signatures, e.g. Synaptogenesis Signaling Pathway, were decreased at D48 (Fig. 3c).

#### Diseases and functions analysis of DEGs

In addition to IPA pathway analysis, diseases and functions analysis was utilized to provide broad biological and pathophysiologic context, with the goal of identifying relative influence or change over time from injury (Fig. 3d). Broad categories are defined by IPA, with further stratification into sub-annotations of specific diseases and functions. In our data set, six categories were identified to have robust longitudinal representation: Cell Death, Inflammatory Response, Cellular Growth and Proliferation, Neurological Disease, Skeletal and Muscular System Development and Function (“Muscle”), and Fibrosis. To deconstruct these specific categories and assimilate into tangible patterns, a part-to-whole approach was employed; the counts of sub-annotations for each broad category, out of sub-annotations for all categories, was calculated per day and represented as a percent. Inflammatory Response predominated the transcriptional landscape across all timepoints until tapering at D48 (Fig. 3d). Representation of Neurologic Disease increased over time, with the highest proportion of representation on D48. Cellular Growth and Proliferation demonstrated an inverse relationship compared with Neurologic Disease, with the highest proportions in the acute and early timepoints (D0–D7) and decreases for the later timepoints. Cell Death was parabolic, with a higher proportion at D0 and then decrease until D7, and then increase for the later timepoints with peak representation by D48.

#### D48 overlapping pathways and critical genes

Because many genes overlap or coordinate in function, we investigated the frequency of overlap of genes considered critical in IPA to provide an assessment of which genes are involved in the interplay of the predominant pathways in our dataset. Described simply, we sought to identify specific genes suspected of participating in the mature neuroma pathology. Collectively, the categories of Inflammatory Response, Neurologic Disease, and Cell Death displayed the highest overlap in gene products at the terminal endpoint (D48) where we have previously demonstrated mature neuroma formation. Therefore, we further examined the degree to which these gene products were shared with multiple categories or were unique to the category in the mature pathophysiologic neuroma (Fig. 3e). Inflammatory Response and Neurologic Disease presented the highest sharing of genes (24.8%), followed by unique expression profiles for Neurologic Disease (23.1%) and Inflammatory Response (21.5%). Cell Death genes were primarily shared with Neurologic Disease (14.9%) or all the represented categories (7.4%). Genes of Neurologic Disease were predominated by ion channel representation (KCNQ2, SCN9A, KCNC1, KCNA4, CHRNA1, GRIA1, NPY2R), whereas Inflammatory Response embodied a mêlée of chemokines/cytokines (CXCL3, CXCR6), immune-specific receptors (CD180, CD22, CD5L), and matrix metalloproteinases (MMP3, MMP12). Finally, key overlapping genes between all 3 categories were those implicated in activated immune responses, such as CD28, CD4, KLRB1, and TYROBP, and also growth factors such as CNTF and TGFA.

### Interaction model to identify genes distinctly significant to rupture injury

To define the cardinal genes associated with neuroma formation, we employed an interaction model to identify the statistically significant genes distinct or influential to the rupture-injury state as compared with the sham injury. Sham injury is an acute inflammatory response associated with the surgical procedure but not neurologic injury, as clearly demonstrated by resolution of gene expression between D7 and D48 and subjection of DEGs to IPA diseases and functions analysis (Supplementary Fig. S1). This approach permits specific identification of pathways associated with the neuroma microenvironment to identify distinctions that might be otherwise too nuanced to appreciate with the overwhelming inflammatory response.

**Figure 4:**
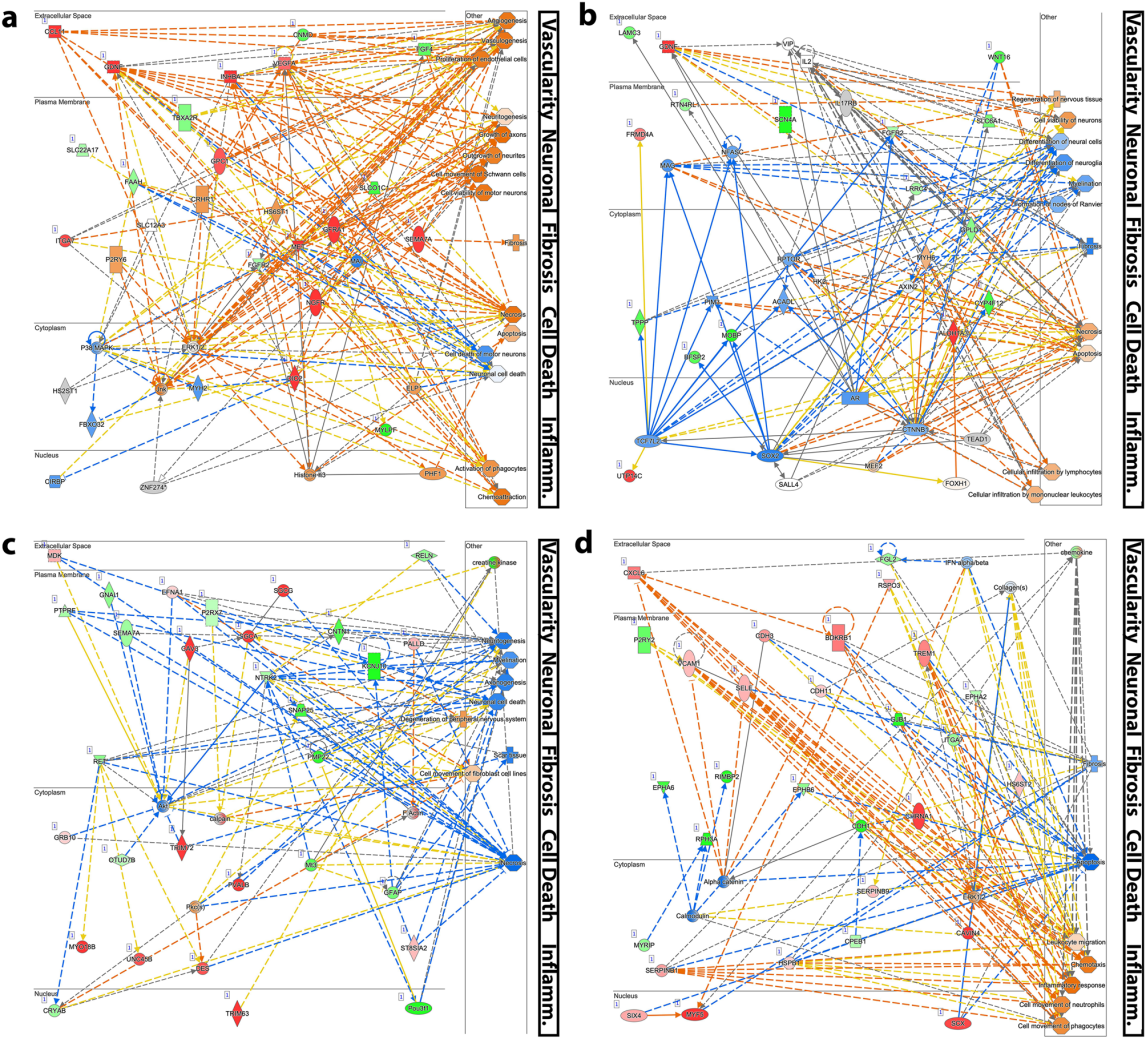
Significant genes distinct to the rupture injury state for (**a**, **b**) day 0 vs. day 2 and (**c**, **d**) day 2 vs. day 7, subjected to Ingenuity network analysis (QIAGEN Inc., https://digitalinsights.qiagen.com/IPA) for construction of de novo genetic networks of highly interconnected genes representative of significant biological function. Overlay with diseases and functions pathways permits elucidation of how these networks are associated to the downstream biological functions. Red represents significant upregulation; green, downregulation; orange, predicted activation; blue, predicted inhibition. Grey indicates the gene is not in the isolated dataset, but identified as highly related per network analysis. Five motifs were identified to which pathways repeatedly align: vascularity, Neuronal, Fibrosis, Cell Death, and Inflammation. Only pathways of a *p*-value < 1 × 10^–5^ are shown and annotated to the identified motifs. (**a**) The top network pathways with predicted activation are largely associated with vascularity (Angiogenesis, Vasculogenesis, Proliferation of Endothelial Cells), neuronal processes (Neuritogenesis, Growth of Axons, Cell Movement of Schwann Cells, Cell Viability of Motor Neurons), fibrosis, and inflammation (Activation of Phagocytes, Chemoattraction), while there is conflicting enrichment of cellular death profiles (Activation: Necrosis, Apoptosis; Inhibition: Neuronal Cell Death). (**b**) The second highest network implicates activation of cell death (Necrosis, Apoptosis) and inflammatory pathways (Cellular Infiltration by Lymphocytes, Cellular Infiltration by Mononuclear Leukocytes), inhibition of fibrosis, and predominately inhibition of neuronal pathways (Differentiation of Neural Cells, Myelination, Formation of Nodes of Ranvier). (**c**) Top network pathways are overwhelmingly inhibitory and align with neuronal fibrosis (Scar tissue), and cell death (Necrosis) motifs. (**d**) The second highest network presents pathways that predominately predict activation of inflammatory pathways, yet inhibition of cell death and fibrosis.

The significant genes determined by the interaction model were then subject to IPA network analysis, which constructs *de-novo* networks based on the interconnectedness of genes in the dataset and the premise that highly interconnected genes reflect germane biological networks. These networks were then independently overlaid with IPA canonical pathways (Supplementary Fig. S2) and IPA diseases and functions (Fig. 4) to provide context of how the de novo*-*constructed genetic networks are related to traditionally established biologic and molecular pathways. Identified pathways from both overlays predominately aligned with 5 motifs: Vascularity, Neuronal, Fibrosis, Cell Death, and Inflammation. Overlaying both IPA canonical pathways and IPA diseases and functions to the network analysis permits independent corroboration of the de novo-constructed networks to downstream biological function.

We focused on IPA diseases and functions analysis as this analysis employs a predication algorithm to determine activation/inhibition of downstream biological function states based on relative contribution of DEGs in our dataset to the genes in the established disease/function pathways (Fig. 4). Only pathways with a *p*-value < 1 × 10^–5^ are presented and annotated to the identified motifs. Furthermore, we selected the transition between D0 and D2 (Fig. 4a,b) and D2 to D7 (Fig. 4c,d) , when the pathways diverged on PCA, thereby focusing on the early events that discriminated outcomes. The top network for D0 vs. D2 (Fig. 4a) largely predicted activation of pathways associated with Vascularity, Neuronal, Fibrosis, and Inflammation. Cell Death paradoxically predicted global activation yet specific inhibition of Neuronal Cell Death. Critical hub genes with significant upregulation included CCL11, GDNF, VEGFA, INHBA, and GFRA1. Significantly downregulated hub genes included FGF4, CNMD, SLCO1C1, and FGFR2. Critical hub genes with predicted inhibition included P38 MAPK, FBXO32, and MAL while predicted activation included JNK, P2RY6, HS6ST1, CHR1, and Histone H3. The second highest network for D0 vs. D2 (Fig. 4b) presented activation of cell death and inflammation pathways and predicted inhibition of fibrosis pathways. Although neuronal pathways presented conflicting activation/inhibition patterns, pathways were largely associated with neuronal inhibition, such as activation of a negative pathway. Critical hub genes with significant upregulation included SLC6A1, SCN4A, WNT16, MOBP, TPPP, BFSP2, and CYP4F12, while significantly downregulated hub genes included GDNF, UTP14C, and ALDH1A3. Critical hub genes with predicted inhibition included SOX2, NFASC, MAG, RPTOR, and CTNNB1, while only MYH6 demonstrated predicted activation. For the top network of D2 vs. D7 (Fig. 4c), we observed reversal of the patterns described on D0 vs. D2; pathways aligning to neuronal, fibrotic, and cell death motifs largely predicted inhibition, while there was limited significant annotation to vascular or inflammatory paths. Critical hub genes with significant upregulation included CAV3, DES, and SGCA. Significantly downregulated hub genes included KCNJ10, PMP22, GFAP, CNTN, and SNAP25. The second top network for D2 vs. D7 (Fig. 4d) largely predicted activation of inflammatory-related paths and inhibition of fibrosis and cell death–related pathways. Critical hub genes with significant activation included SERPINB1, CHRNA1, CXCL6, TREM1, and SCX. Significantly downregulated hub genes included PYRY2, FGL2, EPHB6, EPHA6, and GJB1.

### Immunofluorescence for protein corroboration

#### Structural changes

**Figure 5:**
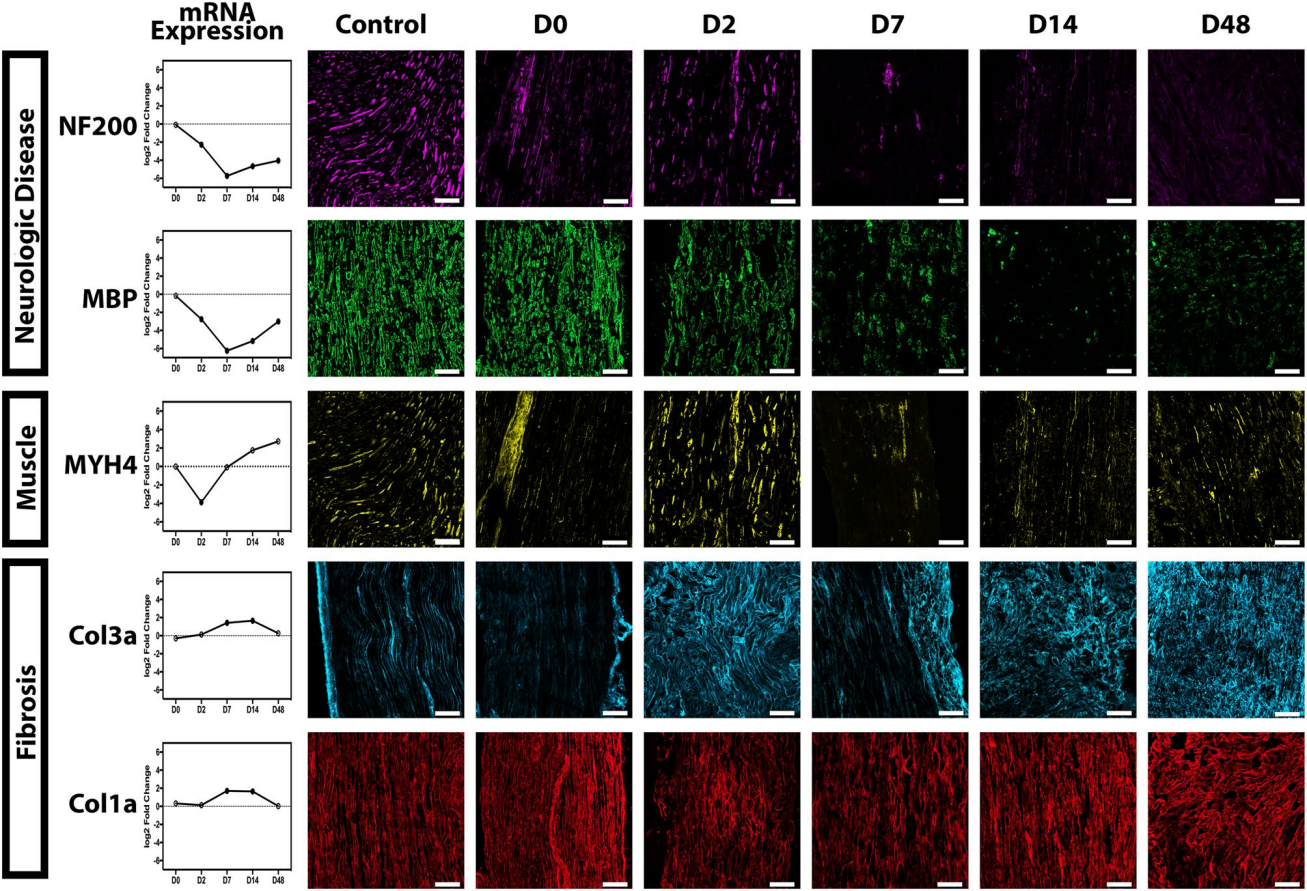
Immunofluorescent corroboration of GSEA and IPA analysis. Longitudinal slices of sciatic nerve were obtained at 10 μm and images acquired at × 200. Log2fold change values from the raw data are presented for transcript to protein comparison. Repeating motifs of neurologic disease, fibrosis, and muscular pathways, were identified through multiple pathway analyses. Neurologic disease was corroborated with axonal (anti-NF200) and myelin sheath (anti-MBP) staining. Expression between these markers is complementary: both demonstrate a dramatic decrease in expression over time. MYH4 is represented in multiple muscle categories, yet as a myosin, has functional multiplicity in the cytoskeleton, which is exemplified by apparent overlap with NF200 at day 0 (D0), D2, and D14, along with control specimens. Fibrosis presents with pathological remodeling as early as 2 days post-injury. Col3a has minute representation in the control nerve, with fluctuations in expression and aberrant deposition over the time course of injury progression. Col1a demonstrates decreased protein expression D2 and D7, followed by increased intensity and disordered remodeling. At D48, both collagens depict intense staining and dearth of microtubule delineation. Scale bar, 50 μm.

Longitudinal and multiple independent genetic analyses identified repeating motifs of Neurologic Disease, Inflammation, Fibrosis, Muscle, Cell Proliferation, and Cell Death. Because these categories were globally represented in GSEA and other IPA analyses, key molecules were assessed through immunofluorescent histology for protein corroboration. Molecules that are often used clinically stood out among the implicated genes in the pathways and thus were used to provide a translational comparison (Fig. 5). The microarchitectural features of Neurologic Disease demonstrated progressive decline in axons (anti-NF200) and myelin (anti-MBP). Thus, inverse correlation between protein expression and representation of neurologic disease in the diseases and functions analysis in Fig. 2 was observed. MYH4 was frequently associated with the muscle pathways yet is also present in the myotubules of many cellular species; thus, the identification of muscle-specific pathways is at risk of misattribution. MYH4 demonstrated apparent overlap with NF200 at D0, D2, and D14 after injury as well as in control samples. Limited expression was observed from D7 to D48 within the nerve. Thus, although the increase in transcript expression could be attributed to small quantities of muscle surrounding the neuroma, which cannot be resected in a gross manner without risking compromising the neuroma itself, immunofluorescent staining suggests MYH4 is common within other cellular origins. Genetic patterns of fibrosis were identified throughout multiple analyses, and aberrant fibrotic deposition is a hallmark of clinical neuroma pathology. In particular, collagen is a well-known component of the endoneurial tubules; thus, we sought to corroborate multiple types in the pathologic progression. Col1a is the predominant collagen of the collagenous fiber type while Col3a is associated with reticular fiber types. By D2, we observed alterations in collagen expression, hallmarked by disorganized matrix deposition that persists longitudinally throughout our timepoints (Fig. 5).

#### Immunologic changes

**Figure 6:**
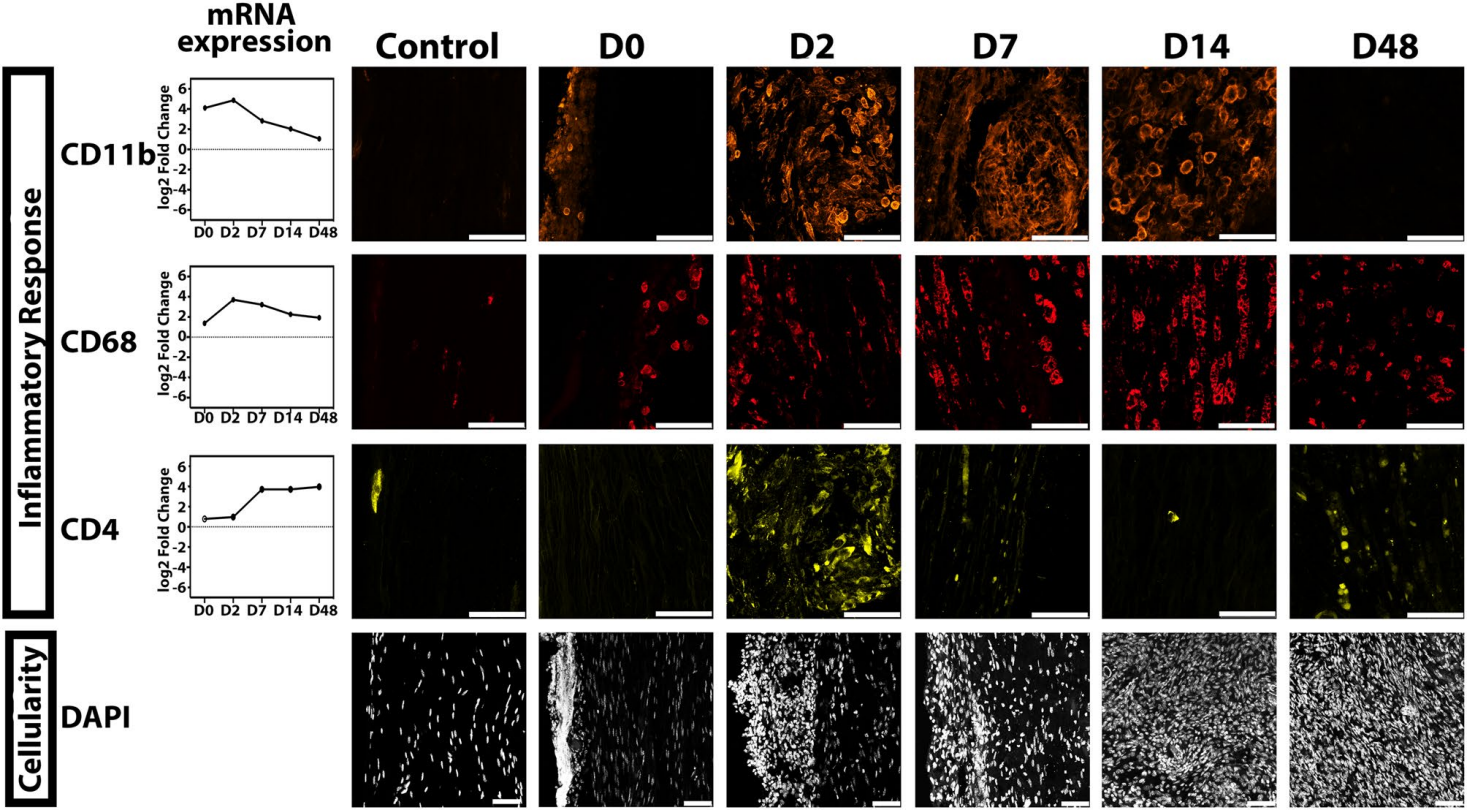
Immunofluorescent corroboration of temporal inflammatory signatures associated with neuroma formation. Immunofluorescence images acquired at × 400 for inflammatory markers and × 200 for DAPI. Cd11b is a pan-granulocyte marker; robust increase in transcript expression is observed acutely after injury, as corroborated by immunofluorescent staining. Expression persists through day 14 (D14), with gradual decline and minute expression by D48. CD68 is a marker for activated macrophages, expression compared with cd11b is delayed with a peak at D2, and gradual decline in transcript expression through D48. An expanded and “foamy” phenotype is observed for D7 and D14 that attenuates with time. Notably, persistence of CD68 is observed in the pathologic neuroma state at the D48 endpoint. CD4 is commonly associated with T-helper cells yet also is a marker for dendritic cells. Pockets of CD4 labeled cells converge with those of cd11b at the early timepoints. In contrast to transcript expression levels, protein corroboration was not observed for D7 and D14. Importantly, we see the reemergence of positive staining at D48, although the phenotype is markedly divergent. DAPI is a nuclear marker and displays a dynamic expression pattern. At our earliest timepoint, intense staining is observed on the exterior of the nerve, notably with undefined nucleic staining. D2 through D7 demonstrate cellular accumulation on the exterior of the nerve, reflected by increased cellularity internally. By D14, cellular infiltration is profound and this hypercellularity persists through D48. Scale bar, 50 μm.

Inflammatory pathways ubiquitously presented across analysis platforms with significant upregulation across timepoints (Fig. 6). CD11b is a pan-granulocyte marker with robust expression after injury—corroborative of the persistent activation of inflammatory paths in the genetic analysis. Pockets of these cells were observed on D2 and D7 that clustered in locations on the exterior of the nerve with some internal infiltration. Surprisingly, robust expression was still observed on D14; although topographically, these cells may exist interior of the formed neuroma, they remained localized to the exterior boundary and were segregated from the apparent dead ends of injured nerve (Supplementary Fig. S3). CD68 presents as a marker for activated M1-like (pro-inflammatory) macrophages, which we observed transition into the nerve by D2, increase in size by D7, develop a “foamy” phenotype at D14, and then decrease in size yet remain in the neuromatous tissue through D48. Interestingly, at this terminal endpoint, CD68 staining appeared fragmented. CD4 is a marker for T-helper cells and dendritic cells in the mouse and thus suggests crosstalk to adaptive immunity. Interestingly, we observed high expression on D2 surrounding the CD11b pockets. Although mRNA expression levels increased by D7 and D14, this did not translate to a functional protein per immunofluorescent staining. At D48, we visualized clusters of CD4 within the neuroma, yet morphologically distinct cells from D2, thus potentially representing a different cell type at this time. DAPI expression appeared to correlate with increased immune cell infiltration. Interestingly, we observed denuclearized DAPI deposited along the exterior of the nerve and dramatic intensity. By D2, robust cellularity was observed on the exterior of the nerve, yet the DAPI largely delineated distinct nuclei when compared with D0. At D7, we observed the infiltration of this cellularity, which persisted through D48.

## Discussion

The regenerative capacity of peripheral nerves is well defined in basic science models, yet failed neurologic recovery continues to plague clinical outcomes. This translational gap may in part be explained by the predominance of animal models investigating mechanisms of successful regeneration. Probing the mechanisms underpinning pathologic nerve regeneration may reveal previously unidentified and underappreciated pathways governing failed regeneration and thereby present as novel targets for therapeutic intervention.

The current investigation sought to further interrogate this pathophysiologic focus by applying RNAseq to identify significantly implicated transcripts and annotate their functional capacity to downstream signaling pathways. To our knowledge, this study is the first transcriptional study of NIC pathophysiology. Here, we have identified inflammatory, cellular death, fibrotic, and neurodegeneration signatures that diverge from pathways identified by traditional models of successful nerve regeneration. These insights afford numerous downstream investigations for future interrogation, with the potential to guide prognostic biomarker heuristics or targeted therapeutic intervention.

### Inflammation

Recently, molecular-level assessments of nerve regeneration and WD have ubiquitously revealed substantial enrichment of pathways associated with inflammation^24–27^. These results corroborate the cellular profiling of seminal WD studies^35–37^; however, the transcriptional profiles depict attenuation of the inflammatory response, as genes specific to inflammatory pathways peak from 4 to 7 days after injury and decline thereafter through day 14, which coincides with onset of signaling pathways associated with axonal guidance, nervous system signaling/plasticity, and nervous system development and function^24,25,27,29^. Most of these studies focus on the timeframe of WD, and only 2 studies to our knowledge have examined timepoints out to 4 weeks after injury, reporting a single pathway significantly associated with inflammation at later timepoints, consistent with the interpretation that inflammation is successfully resolving^25,38^.

By contrast, our neuroma model provides evidence of persistently activated inflammatory genetic programs. Multiple IPA analyses and GSEA indicate a peak in inflammatory activity at D2 after injury and sustained longitudinal activation though D48. Inflammatory pathway analysis was corroborated by markers of both innate and adaptive immunity, such as CD11b, CD68, and CD4. CD11b, a pan-myeloid marker, demonstrated persistent presence of both mRNA transcripts and histologic evidence at 14 days post-injury—a timepoint traditionally associated with the culmination of WD and inflammatory resolution in recoverable crush injury models^39,40^. The cellular phenotype potentially indicates chronic myeloid cell recruitment to or *de-novo* proliferation within the neuroma microenvironment, as it is unlikely for these cells to be long-lived^41,42^. CD68 is predominately associated with activated, phagocytic macrophages, while CD4+ cells traditionally annotate T helper cells; both demonstrated prolonged presence in the mature neuroma. Furthermore, transcriptional activity failed to resolve towards the homeostatic levels of control nerves, while DAPI revealed profound hypercellularity of the neuroma.

To our knowledge, no other studies have examined a protracted timepoint (7 weeks post-injury) and associated nerve-specific genetic activity profile. This terminal endpoint is more faithful to the timeline of the clinical disease course and surgical repair, and clinical neuroma specimens similarly note inflammatory cell presence at prolonged timepoints after injury^9–11^. Persistent activation of inflammatory pathways is also a common transcriptional correlate of neuropathic pain in higher-order structures (brain^17,43^, spinal cord^44,45^, and dorsal root ganglia^46,47^) after peripheral nerve injury. Importantly, although the adaptive immune response to nerve injury is incompletely characterized, previous studies suggest the adaptive immune cells have little impact on the kinetics of WD, and suppression may even may facilitate peripheral nerve regeneration^48,49^. Conversely, aberrant activation of adaptive immunity is implicated in chronic pain states, potentially governing steady recruitment of inflammatory species thereby influencing axonal sensitization^50^. Therefore, the persistent activity of the adaptive immune system may be a critical event in neuroma formation and neuropathic pain pathogenesis in the nerve microenvironment.

Resolution of the inflammatory response is widely accepted as the precursor to proper wound healing to permit coordinated architectural remodeling^51^. This process is largely attributed to phagocytosis of dead and dying cells in a process known as efferocytosis^52^. Clearance of dead and dying cells removes cells secreting Damage-associated Molecular Patterns (DAMPs), cytokines, and chemokines—molecules that propagate the inflammatory response and are implicated in contributing to neuropathic pain^53^. In SCI, the self-limited nature of the inflammatory response is corrupted, and sustained activation is associated with fibrotic scar development and secondary pathogenesis^13,54^. This environment is paralleled in the pathophysiologic peripheral neuroma^39,55^, suggesting that aberrant inflammatory activation or failed resolution may similarly contribute to a microenvironment inhibitory to successful regeneration^56,57^. While synergistic cooperation between immune and nervous systems is critical for successful nerve regeneration, our data indicate an unmitigated presence of inflammation, consistent with histology from human specimens^11^, suggesting that the nature of the inflammatory response may be critical in pathophysiologic neuroma formation.

### Cellular death

Although interrogation of neuronal cellular death profiles in the spinal cord and dorsal root ganglia has been widely studied, investigation of cellular death cascades at the level of the nerve has been limited^39,58,59^. Notably, there is scant annotation of cellular death pathways in transcriptional studies of favorable regeneration^28,60–62^. In our study, cellular death pathways demonstrated bimodal activation across multiple timepoints and persisted in the mature neuroma microenvironment 48 days after injury. Given that this study is at the level of the nerve, our results capture a death turnover of cells in the neuroma microenvironment, which could include resident immune, glial, or fibrotic cell lineages in addition to hematogenous immune cell influx. This evidence is corroborated by the interaction model, which specifically predicts inhibition of neuronal cell death, despite implicating activation of apoptotic and necrotic pathways acutely after injury (D0 and D2). Thereafter, overall cell death is suppressed at D7, which coincides with additional inflammatory recruitment and the downregulation of neuronal function. Finally, increased annotation to cell death pathways persists through the remaining timepoints and overlaps with sustained activation of neurodegenerative and inflammatory pathways in the mature neuroma environment.

Cellular death pathways are dually implicated in antagonizing or abrogating inflammatory cascades; apoptosis and efferocytosis promote inflammatory resolution while necroptosis and pyroptosis prime a proinflammatory environment^63,64^. Recent work in a model of sciatic nerve transection has demonstrated dysregulated macrophage cell death in the form of pyroptosis biases the cytokine profile towards a more pro-inflammatory microenvironment. When pyroptosis is specifically ablated in macrophages, clearance of apoptotic cells is enhanced and the pro-inflammatory microenvironment is reversed, which translated to increased axonal regeneration and improved functional recovery^58^. Similarly, in SCI, secondary pathogenesis is associated with activation of numerous cell death pathways and exacerbation of the inflammatory response. Consequently, targeted inhibition attenuates the inflammatory response and improves neurologic recovery profiles; thus, a proposed neuroprotective strategy involves targeted inhibition of cellular death pathways to abrogate pathophysiologic sequelae^64–66^. Although further mechanistic study is warranted to elaborate specific cellular death pathways in peripheral nerve trauma (e.g., necroptosis vs. apoptosis), our data suggest coordinated signatures of WD may be perturbed by cell death dysregulation and contribute to neuroma formation in the pathophysiologic scenario.

### Fibrosis

The canonical hallmark of neuroma is the profound abundance of fibrosis that enlarges and swells the nerve; however, it remains in doubt whether the fibrosis is causal or sequela to the failure for regeneration^67^. In the studies examining transcriptional correlates of successful regeneration, identification of fibrotic transcripts has been limited, meaning annotation to downstream biological pathways is also limited^26,27^. Single-cell RNAseq has identified fibroblasts and phenotype derivatives within the homeostatic and regenerating peripheral nerve, but, because these models do not develop neuromas, enriched fibrosis is not expected^68–70^.

In our pathophysiologic neuroma model, we have identified contribution to fibrogenesis in multiple independent transcriptional analyses, which were corroborated by robust chaotic collagen deposition in our histologic assays. We suspect that an even more pronounced attribution to these pathways may be present yet many fibrotic pathways are latent within broad disease states (e.g., cancer, cystic fibrosis). Although extracellular matrix proteins are necessary for reestablishing the endoneurial tubules that provide the nerve mechanical stability and continuity to the distal target organs, aberrant and uncoordinated fibrosis is implicated in inhibiting axonal growth^71^. These events are recapitulated in SCI, where scar-free healing is associated with improved axonal regeneration^72^; this provides a rationale that the prevention of scar formation may be a viable mechanistic target for therapeutic intervention.

### Neurodegeneration and metabolism

Transcriptional analyses of peripheral nerve injury in successful models of regeneration have highlighted pathways of neuronal regeneration and Schwann cell activation^25–27^. In our model, we conversely observed downregulation of similar paths, which contrasted against the immense activation of the inflammatory, cell death, and fibrotic pathways. Metabolic pathways were significantly downregulated across all timepoints, which may correlate to dysregulated Schwann cell activity, as prior research has identified that glycolytic shifts support axonal regeneration^73^. Interestingly, the enriched transcripts specific to the rupture injury state predict activation of neuronal and Schwann cell activity acutely after injury. Yet by day 7, these efforts are reversed and inhibited, suggesting a critical timepoint at which genetic programs may switch towards pathophysiologic regeneration. This neuronal inhibition coincides with enriched inflammatory signatures distinct from those of surgical manipulation, as indicated by the bifurcation of transcriptional clusters between sham and rupture injury. Collectively, these insights suggest that manipulation of the microenvironment at this critical timepoint could counteract the transition towards pathophysiologic neuroma formation.

The role of metabolic processes in regeneration and immunologic reprograming is gaining intellectual traction^74^. Metabolic pathways such as fatty acid biosynthesis and lipid metabolism are notably downregulated after spinal cord injury, and targeted inhibition is associated with decreased axonal regeneration in vitro. Similar downregulation was not observed in a peripheral nerve transection (recoverable) nerve injury model^75^. A recent body of work has used single-cell RNAseq to elaborate the reprogramming of macrophage metabolism from predominately glycolytic to oxidative phosphorylation in a recovering nerve injury model (crush). This metabolic transition is associated with a proinflammatory to inflammatory resolution phenotype in macrophages and is observed about day 7 after injury^76^. Interestingly, metabolic cycles have similarly been implicated in inflammatory pain phenotypes; reduced oxidative phosphorylation is associated with the apogee of inflammatory-associated hyperalgesia whereas activation of oxidative phosphorylation resolves this pain pathogenesis^77^. Collectively, there are interesting metabolic parallels between our pathophysiologic peripheral nerve injury model, comorbidities such as chronic pain, and the recalcitrant regeneration programs of the central nervous system after injury, which contrasts against the metabolic activity of recovering nerve injury models.

### Translational impact: targets for therapeutic intervention

As there are currently no therapeutic interventions to prevent NIC formation, the pathways identified may afford further insight for scientific interrogation and opportunities for clinical translation. Given the intricate coordination among the inflammatory, cellular death, and fibrotic pathways, targeting their genesis may be a most promising avenue. For example, we know efferocytosis is critical for inflammatory resolution and that failure to coordinate the immune response can lead to excessive fibrotic deposition^51,52^. Therefore, efforts to explore how the immune response resolves in pathophysiologic nerve injury, such as identifying whether local neutrophil apoptosis occurs or detailing the events governing immune cell egress, are promising next steps.

However, a common clinical challenge is intervention at an appropriate timepoint after injury, which may not lend itself to immunomodulation efforts at the genesis of the inflammatory response. Therefore, a multipronged approach of acute, middle, or late interventions may provide more clinically feasible strategies. For example, a mid-term approach may center on shifting immune cells towards inflammatory resolution by promoting oxidative phosphorylation. Such techniques have already been applied to preclinical models of CNS injury, where studies manipulating the bioenergetic cascades demonstrate improved axonal regeneration and functional recovery in SCI^78,79^. Efforts to understand metabolic pathways in successful models of peripheral nerve regeneration have similarly demonstrated favorable outcomes, yet metabolism remains unexplored in pathophysiologic peripheral nerve regeneration^73,80^. Therefore, metabolic reprogramming to dually promote inflammatory resolution and redirect the microenvironment towards a more proregenerative state is a feasible strategy to overcome temporal restrictions in the clinical population.

Fibrosis is a cardinal feature of neuroma formation and, as such, lends itself as a viable strategy for more protracted injuries where manipulation of the inflammatory response may be a belated intervention. For example, HSP47 is critical for collagen production and is upregulated in a variety of tissues (including nerve) after injury^81,82^. HSP47 inhibition, through both siRNA knockdown and knockout models, reduces fibrosis and scar formation in liver and peritoneum^83–85^. Therefore, translation to peripheral nerve trauma may similarly decrease the fibrotic pathogenesis of neuroma formation and improve regenerative outcomes. Alternatively, application of low-concentration enzymes, which break down collagen (e.g., collagenase), may locally help degrade collagen of the neuroma while preserving the functionality of other cells critical to regeneration, such as perineurial glia and Schwann cells^86,87^. This strategy has already been approved by the US Food and Drug Administration for Dupuytren contracture, another disease hallmarked by unmitigated collagen deposition, with recent efforts focused on creating sustained delivery strategies for the gradual release of collagenase^88^. Collectively, a singular intervention may not suffice for the treatment of traumatic peripheral nerve injuries, and a multidimensional, temporal approach may provide the most translatable intervention from the transcriptional signatures identified in our discovery science model.

## Limitations

Applying restrictions to differentially expressed genes selects for those that are mathematically significant. Pragmatically, genes with transcriptional levels less than our fold change or significance thresholds could be biologically germane to neuroma formation, and this analysis may fail to capture these biological nuances. We additionally restricted our analysis to protein coding transcripts. Although recent efforts have revealed pivotal roles for noncoding RNAs in the regulation of transcriptional activity, these were not a focus of the current study given there is limited pathway annotation of this knowledge in the analysis software. Finally, bulk RNAseq cannot distinguish to which cell types the transcripts may belong, and it is also difficult to draw direct comparisons to other studies given the heterogeneity of sequencing platforms and bioinformatic analysis pipelines.

## Conclusion

Collectively, through transcriptional profiling and histologic validation, we identified significant genetic activity annotated to longitudinal activation of the inflammatory, cellular death, and fibrotic pathways in our pathophysiologic neuroma model. These three processes are intrinsically entwined; inflammation resolves through coordinated cellular death processes, which orchestrate an environment for concerted regeneration as opposed to scar formation. Antithetically, activation of this triad is coupled with persistent downregulation of neuronal and myelination pathways, which are the focus of recoverable nerve injury models. These insights afford a novel lens through which the scientific and clinical community can approach therapeutic manipulation of the immune, metabolic, cellular death, and fibrotic processes to prevent aberrant activity and promote the intrinsic regenerative capacity of the peripheral nervous system.

## Methods

### Nerve injury and tissue processing

Sixty-one male C57BL/6 J mice aged 8–10 weeks were randomly assigned to groups: days 0 (6 h post-injury), 2, 7, 14, and 48 for both sham (n = 6) and rupture injuries (n = 5). Control nerves were harvested where no surgical intervention was performed (n = 6). For sham and control samples, 2 biological replicates of 3 nerves were used to obtain sufficient RNA quantity for library preparation. All experiments were approved by the Institutional Animal Care and Use Committee at the University of Utah (Protocol #19-04,007) and performed in accordance with relevant guidelines and regulations. The study is reported per the recommendations detailed by the ARRIVE guidelines.

Surgery and injury were performed as previously reported^33,34^. In brief, animals were anesthetized with isoflurane, and using a dorsal approach, the left hindlimb was opened from knee to sciatic notch via blunt dissection to delicately resect the sciatic nerve from surrounding fascia. For the rapid-stretch rupture injury, weight drop applied to a pulley/hook system permitted precise displacement of nerve^31^. After careful placement of nerve on the hook, stretch rupture was obtained with a stretch excursion of > 15 mm while sham-injured nerves remained on the hook for an equivalent time (~ 30 s). Ruptured nerves were repositioned back into the appropriate anatomical area without interventional coaptation, and the incision was closed with nylon suture. Postoperatively, animals were administered bupivacaine (2 mg/kg, topical) and carprofen (5 mg/kg, subcutaneous) for pain control and enrofloxacin (5 mg/kg, subcutaneous) for infection prevention.

### Sample preparation

Nerves were harvested at the predefined timepoints from sciatic notch to distal trifurcation (15 mm), mechanically homogenized in Trizol reagent (Life Technologies, Carlsbad, CA), and stored at − 80 °C until further processing. Control nerves were harvested immediately from euthanized animals that were subject to no experimental intervention. The sample was then mixed with chloroform (1:4 ratio) to produce an aqueous phase containing the total RNA, which was subsequently isolated and used in all further steps. To isolate total RNA for sequencing, the sample was processed through an RNeasy Mini column (QIAGEN, Valencia, CA) per manufacturer instructions. The concentration and quality of isolated RNA were determined by sampling through Agilent ScreenTape Assay (Agilent Technologies, Santa Clara, CA), and only those with an RNA integrity number ≥ 7.0 proceeded for library preparation. Next-generation RNAseq was performed with the Illumina NovaSeq6000 (San Diego, CA) with 2 × 50 base pair sequences and 25 million read-pairs per sample. Sample preparation, sequencing, and further analysis was performed in a blinded fashion.

### Bioinformatics analysis

Raw reads derived from sequencing were optimized, trimmed, and aligned to the reference database using STAR software in two-pass mode to output a binary alignment map file sorted by coordinates. Mapped reads were assigned to annotated genes using featureCounts version 1.6.3 (http://subread.sourceforge.net/)^89^. DEGs for each injury grade were identified using a 5% false discovery rate with DESeq2 version 1.30.0 (https://bioconductor.org/packages/release/bioc/html/DESeq2.html) and log2fold change compared with control expression levels^90^. Global expression profiles were examined through graphing DEG activity, cluster analysis, principal component (PCA) and interaction model analyses. DEG activity was graphed using GraphPad Prism version 6.0.0 for Mac (GraphPad Software, San Diego, California USA, www.graphpad.com). For the cluster analysis, samples were hierarchically clustered using a Euclidean distance matrix of regularized log-transformed counts as input (RStudio 4.2.1: pheatmap 1.0.12. https://cran.r-project.org/web/packages/pheatmap/index.html). PCA was performed with RStudio 4.2.1: highcharter 0.9.4 (https://cran.r-project.org/web/packages/highcharter/index.html). For the interaction model, we combined injury and day into a single group and identified differentially expressed genes using a 5% false discovery rate with DESeq2 version 1.30.1^90^. In addition, to check whether the injury effect differs across days, we also used an interaction model with injury × day in the design formula. This permitted identification of statistically significant genes distinct to the rupture-injured state for comparison at timepoints D0 vs. D2 and D2 vs. D7 where PCA Euclidian distance mapping suggested similar cluster profiles for rupture and sham injuries.

Critical pathways associated with pathological remodeling were identified using GSEA (version 4.1.0, Broad Institute, San Diego, CA) and IPA (QIAGEN Inc., https://digitalinsights.qiagen.com/IPA). Independent analyses were employed to provide an additional layer of validation. GSEA was performed using pre-ranked RNA differential expression data from DESEQ and ENSEMBL hallmarks and chip platforms, resulting in longitudinal comparisons of up- and downregulated gene sets between rupture and control^91^. For IPA, differentially expressed genes were uploaded into the IPA software, and core analysis was run using a *p*-value < 0.05 and fold change > 2 or <−2 threshold to identify analysis-ready molecules. These molecules were then overlaid with the Ingenuity pathway knowledge base (IPKB), which employs a right-tailed Fischer’s exact test to identify statistically significant canonical signaling pathways and biological functional activity associated with the gene set. IPA network analysis was also implemented to identify de novo gene network signatures. This analysis employs the assumption that highly interconnected interactions are likely reflective of considerable biological function and thus may reveal functions that may not be captured in traditional, a priori analyses (such as the canonical pathways or diseases and functions)^92^. Additionally, the networks generated can also be overlaid with either diseases and functions or canonical pathways to provide context of how the de novo-constructed genetic networks are related to traditionally established biologic and molecular pathways.

### Histologic validation

A separate cohort of animals was subjected to nerve injury as described above for corroborative immunofluorescence histology on proteins of interest regarding pathways and cell populations identified in the RNAseq analyses. Specifically, we sought to corroborate the repeating motifs of Neurologic Disease, Skeletal and Muscular System Development and Function (abbreviated “Muscle”), Fibrosis, Inflammatory Response, and Cellularity.

Timepoints were determined a priori with an n > 5 per timepoint (total n = 34). Upon harvest, nerves were placed in 4% paraformaldehyde for 30 min then transferred to 30% sucrose overnight before embedding in OCT compound, frozen, and stored at − 80 °C. Longitudinal slices were acquired at 10 μm, and corroborative immunofluorescent staining was performed for genes identified in paths of both GSEA and IPA analyses.

To visualize the microarchitectural features of neurologic disease transcripts, myelin (anti-MBP, 1:500, ab123500, Abcam, Cambridge, UK) and axons (anti-NF200, 1:500, N0142 (N52), Sigma, St. Louis, MO) were examined. Myosin (anti-MYH4 1:1000, Sigma M7523) was stained to corroborate the Muscle category. Pathways implicating fibrosis were validated with staining for collagen1a (anti-Col1a, 1:800, Abcam ab34710), the major collagen of collagenous fibers, and collagen3a (anti-ER-TR7 1:500, Abcam ab51824), the predominant collagen of reticular fibers. All primaries were paired with appropriate secondaries. Images were acquired at × 200 on confocal microscope (Zeiss LSM 800, Jena, Germany) and processed using Zeiss Zen 2.3 software. For the inflammatory response, pan-granulocyte staining against ITGAM (anti-CD11b 1:500, Abcam ab8878), phagocytic proinflammatory macrophages (anti-CD68, 1:500, NBP2-33,337, Novus Biologicals, Littleton, CO), and pan helper T-cell (anti-CD4 1:100, Abcam ab183685) were used as corroborative evidence for transcripts implicated in overlapping pathways, with images acquired at × 400. Finally, cellularity, with particular emphasis on the exterior of the nerve, was optimized to nuclei with DAPI (4′,6-diamidino-2-phenylindole) staining and acquired at × 200.

## Supplementary Information

Supplementary Information.

## Data Availability

The datasets used and/or analyzed during the current study available from the corresponding author on reasonable request.

## References

[1] Lundborg G (2000). A 25-year perspective of peripheral nerve surgery: Evolving neuroscientific concepts and clinical significance. J Hand Surg.

[2] Dubuisson AS, Kline D (2002). Brachial Plexus injury: A survey of 100 consecutive cases from a single service. Neurosurgery.

[3] Tedeschi A, Bradke F (2017). Spatial and temporal arrangement of neuronal intrinsic and extrinsic mechanisms controlling axon regeneration. Curr Opin Neurobiol.

[4] Mahar M, Cavalli V (2018). Intrinsic mechanisms of neuronal axon regeneration. Nat Rev Neurosci.

[5] Chen L, Gao SC, Gu YD, Hu SN, Xu L, Huang YG (2008). Histopathologic study of the neuroma-in-continuity in obstetric brachial plexus palsy. Plast Reconstr Surg.

[6] Oliveira KMC, Pindur L, Han Z, Bhavsar MB, Barker JH, Leppik L (2018). Time course of traumatic neuroma development. PLoS ONE.

[7] Cravioto H, Battista A (1981). Clinical and ultrastructural study of painful neuroma. Neurosurgery.

[8] Sunderland SS (1990). The anatomy and physiology of nerve injury. Muscle Nerve.

[9] Vora AR, Bodell SM, Loescher AR, Smith KG, Robinson PP, Boissonade FM (2007). Inflammatory cell accumulation in traumatic neuromas of the human lingual nerve. Arch Oral Biol.

[10] Egami S, Tanese K, Honda H, Kasai H, Yokoyama T, Sugiura M (2016). Traumatic neuroma on the digital tip: Immunohistochemical analysis of inflammatory signaling pathways. J Dermatol.

[11] Mahan MA, Abou-Al-Shaar H, Karsy M, Warner W, Yeoh S, Palmer CA (2019). Pathologic remodeling in human neuromas: Insights from clinical specimens. Acta Neurochir (Wien).

[12] Bradbury EJ, Burnside ER (2019). Moving beyond the glial scar for spinal cord repair. Nat Commun.

[13] Kwiecien JM, Dabrowski W, Dąbrowska-Bouta B, Sulkowski G, Oakden W, Kwiecien-Delaney CJ (2020). Prolonged inflammation leads to ongoing damage after spinal cord injury. PLoS ONE.

[14] Levy SE, Myers RM (2016). Advancements in next-generation sequencing. Annu Rev Genomics Hum Genet.

[15] Li H, Wan HQ, Zhao HJ, Luan SX, Zhang CG (2019). Identification of candidate genes and miRNAs associated with neuropathic pain induced by spared nerve injury. Int J Mol Med.

[16] Tajerian M, Alvarado S, Millecamps M, Vachon P, Crosby C, Bushnell MC (2013). Peripheral nerve injury is associated with chronic, reversible changes in global DNA methylation in the mouse prefrontal cortex. PLoS ONE.

[17] Cai G, Zhu Y, Zhao Y, Chen J, Guo C, Wu F (2020). Network analysis of miRNA and mRNA changes in the prelimbic cortex of rats with chronic neuropathic pain: Pointing to inflammation. Front Genet.

[18] Zhou J, Fan Y, Chen H (2017). Analyses of long non-coding RNA and mRNA profiles in the spinal cord of rats using RNA sequencing during the progression of neuropathic pain in an SNI model. RNA Biol.

[19] Weng J, Li DD, Jiang BG, Yin XF (2020). Temporal changes in the spinal cord transcriptome after peripheral nerve injury. Neural Regen Res.

[20] Du H, Shi J, Wang M, An S, Guo X, Wang Z (2018). Analyses of gene expression profiles in the rat dorsal horn of the spinal cord using RNA sequencing in chronic constriction injury rats. J Neuroinflammation.

[21] Sun W, Kou D, Yu Z, Yang S, Jiang C, Xiong D (2020). A Transcriptomic analysis of neuropathic pain in rat dorsal root ganglia following peripheral nerve injury. Neuromolecular Med.

[22] Li S, Xue C, Yuan Y, Zhang R, Wang Y, Wang Y (2015). The transcriptional landscape of dorsal root ganglia after sciatic nerve transection. Sci Rep.

[23] Wu S, Marie Lutz B, Miao X, Liang L, Mo K, Chang YJ (2016). Dorsal root ganglion transcriptome analysis following peripheral nerve injury in mice. Mol Pain.

[24] Xing L, Cheng Q, Zha G, Yi S (2017). Transcriptional profiling at high temporal resolution reveals robust immune/inflammatory responses during rat sciatic nerve recovery. Mediators Inflamm..

[25] Yu J, Gu X, Yi S (2016). Ingenuity pathway analysis of gene expression profiles in distal nerve stump following nerve injury: Insights into wallerian degeneration. Front Cell Neurosci.

[26] He B, Pang V, Liu X, Xu S, Zhang Y, Djuanda D (2021). Interactions among nerve regeneration, angiogenesis, and the immune response immediately after sciatic nerve crush injury in Sprague-Dawley rats. Front Cell Neurosci.

[27] Guo Q, Zhu H, Wang H, Zhang P, Wang S, Sun Z (2018). Transcriptomic landscapes of immune response and axonal regeneration by integrative analysis of molecular pathways and interactive networks post-sciatic nerve transection. Front Neurosci.

[28] Han D, Chen Y, Kou Y, Weng J, Chen B, Yu Y (2016). Profiling of the dynamically altered gene expression in peripheral nerve injury using NGS RNA sequencing technique. Am J Transl Res.

[29] Barrette B, Calvo E, Vallières N, Lacroix S (2010). Transcriptional profiling of the injured sciatic nerve of mice carrying the Wld(S) mutant gene: Identification of genes involved in neuroprotection, neuroinflammation, and nerve regeneration. Brain Behav Immun.

[30] Palispis WA, Gupta R (2017). Surgical repair in humans after traumatic nerve injury provides limited functional neural regeneration in adults. Exp Neurol.

[31] Mahan MA, Yeoh S, Monson K, Light A (2019). Rapid stretch injury to peripheral nerves: Biomechanical results. Clin Neurosurg.

[32] Warner WS, Yeoh S, Light A, Zhang J, Mahan MA (2020). Rapid-stretch injury to peripheral nerves: Histologic results. Clin Neurosurg.

[33] Mahan MA, Warner WS, Yeoh S, Light A (2020). Rapid-stretch injury to peripheral nerves: Implications from an animal model. J Neurosurg.

[34] Yeoh S, Warner WS, Eli I, Mahan MA (2020). Rapid-stretch injury to peripheral nerves: Comparison of injury models. J Neurosurg.

[35] Waller AV (1850). Experiments on the section of glossopharyngeal and hypoglossal nerves of the frog and observations of the alternatives produced thereby in the structure of their primitive fibre. Philos Trans R Soc Lond.

[36] Avellino AM, Hart D, Dailey AT, MacKinnon M, Ellegala D, Kliot M (1995). Differential macrophage responses in the peripheral and central nervous system during wallerian degeneration of axons. Exp Neurol.

[37] Barrette B, Hébert MA, Filali M, Lafortune K, Vallières N, Gowing G (2008). Requirement of myeloid cells for axon regeneration. J Neurosci.

[38] Cheng Q, Wang YX, Yu J, Yi S (2017). Critical signaling pathways during wallerian degeneration of peripheral nerve. Neural Regen Res.

[39] Kalinski AL, Yoon C, Huffman LD, Duncker PC, Kohen R, Passino R (2020). Analysis of the immune response to sciatic nerve injury identifies efferocytosis as a key mechanism of nerve debridement. Elife.

[40] Kobayashi D, Kiguchi N, Saika F, Kishioka S, Matsuzaki S (2020). Insufficient efferocytosis by M2-like macrophages as a possible mechanism of neuropathic pain induced by nerve injury. Biochem Biophys Res Commun.

[41] Patel AA, Ginhoux F, Yona S (2021). Monocytes, macrophages, dendritic cells and neutrophils: An update on lifespan kinetics in health and disease. Immunology.

[42] Hidalgo A, Chilvers ER, Summers C, Koenderman L (2019). The neutrophil life cycle. Trends Immunol.

[43] Alvarado S, Tajerian M, Millecamps M, Suderman M, Stone LS, Szyf M (2013). Peripheral nerve injury is accompanied by chronic transcriptome-wide changes in the mouse prefrontal cortex. Mol Pain.

[44] Jeong H, Na YJ, Lee K, Kim YH, Lee Y, Kang M (2016). High-resolution transcriptome analysis reveals neuropathic pain gene-expression signatures in spinal microglia after nerve injury. Pain.

[45] Niehaus JK, Taylor-Blake B, Loo L, Simon JM, Zylka MJ (2021). Spinal macrophages resolve nociceptive hypersensitivity after peripheral injury. Neuron.

[46] Yu X, Liu H, Hamel KA, Morvan MG, Yu S, Leff J (2020). Dorsal root ganglion macrophages contribute to both the initiation and persistence of neuropathic pain. Nat Commun.

[47] Ray P, Torck A, Quigley L, Wangzhou A, Neiman M, Rao C (2018). Comparative transcriptome profiling of the human and mouse dorsal root ganglia: An RNA-seq-based resource for pain and sensory neuroscience research. Pain.

[48] Cashman CR, Hoke A (2017). Deficiency of adaptive immunity does not interfere with Wallerian degeneration. PLoS ONE.

[49] Bombeiro AL, Santini JC, Thomé R, Ferreira ERL, Nunes SLO, Moreira BM (2016). Enhanced immune response in immunodeficient mice improves peripheral nerve regeneration following axotomy. Front Cell Neurosci.

[50] Kanashiro A, Hiroki CH, da Fonseca DM, Birbrair A, Ferreira RG, Bassi GS (2020). The role of neutrophils in neuro-immune modulation. Pharmacol Res.

[51] Ayala A, Chung CS, Grutkoski PS, Song GY (2003). Mechanisms of immune resolution. Crit Care Med.

[52] Sugimoto MA, Vago JP, Perretti M, Teixeira MM (2019). Mediators of the resolution of the inflammatory response. Trends Immunol.

[53] Ellis A, Bennett DLH (2013). Neuroinflammation and the generation of neuropathic pain. Br J Anaesth.

[54] Prüss H, Kopp MA, Brommer B, Gatzemeier N, Laginha I, Dirnagl U, Schwab JM (2011). Non-resolving aspects of acute inflammation after spinal cord injury (SCI): Indices and resolution plateau. Brain Pathol.

[55] Kuhlmann T, Bitsch A, Stadelmann C, Siebert H, Brück W (2001). Macrophages are eliminated from the injured peripheral nerve via local apoptosis and circulation to regional lymph nodes and the spleen. J Neurosci.

[56] McLean NA, Verge VMK (2016). Dynamic impact of brief electrical nerve stimulation on the neural immune axis—polarization of macrophages toward a pro-repair phenotype in demyelinated peripheral nerve. Glia.

[57] Braga TT, Agudelo JSH, Camara NOS (2015). Macrophages during the fibrotic process: M2 as friend and foe. Front Immunol.

[58] Tao Y, Wang F, Xu Z, Lu X, Yang Y, Wu J (2021). Gasdermin D in peripheral nerves: The pyroptotic microenvironment inhibits nerve regeneration. Cell Death Discov.

[59] Hart AM, Terenghi G, Wiberg M (2008). Neuronal death after peripheral nerve injury and experimental strategies for neuroprotection. Neurol Res.

[60] Lu M, Cheng Q, Fang Z, Yi S (2018). Analysis of biological functional networks during sciatic nerve repair and regeneration. Mol Cell Biochem.

[61] Jiang N, Li H, Sun Y, Yin D, Zhao Q, Cui S, Yao D (2014). Differential gene expression in proximal and distal nerve segments of rats with sciatic nerve injury during Wallerian degeneration. Neural Regen Res.

[62] Zhang Y, Zhan Y, Han N, Kou Y, Yin X, Zhang P (2017). Analysis of temporal expression profiles after sciatic nerve injury by bioinformatic method. Sci Rep.

[63] Kim EH, Wong SW, Martinez J (2019). Programmed necrosis and disease: we interrupt your regular programming to bring you necroinflammation. Cell Death Differ.

[64] Kolb JP, Oguin TH, Oberst A, Martinez J (2017). Programmed cell death and inflammation: Winter is coming. Trends Immunol.

[65] Alizadeh A, Dyck SM, Karimi-Abdolrezaee S (2019). Traumatic spinal cord injury: An overview of pathophysiology, models and acute injury mechanisms. Front Neurol.

[66] Shi Z (2021). Programmed cell death in spinal cord injury pathogenesis and therapy. Cell Prolif.

[67] Kline DG, Nulsen FE (1972). The neuroma in continuity: Its preoperative and operative management. Surg Clin North Am.

[68] Carr MJ, Toma JS, Johnston APW, Steadman PE, Yuzwa SA, Mahmud N (2019). Mesenchymal precursor cells in adult nerves contribute to mammalian tissue repair and regeneration. Cell Stem Cell.

[69] Gerber D, Pereira JA, Gerber J, Tan G, Dimitrieva S, Yángüez E, Suter U (2021). Transcriptional profiling of mouse peripheral nerves to the single-cell level to build a sciatic nerve atlas (Snat). Elife.

[70] Chen B, Banton MC, Singh L, Parkinson DB, Dun XP (2021). Single cell transcriptome data analysis defines the heterogeneity of peripheral nerve cells in homeostasis and regeneration. Front Cell Neurosci.

[71] Fertala J, Rivlin M, Wang ML, Beredjiklian PK, Steplewski A, Fertala A (2020). Collagen-rich deposit formation in the sciatic nerve after injury and surgical repair: A study of collagen-producing cells in a rabbit model. Brain Behav.

[72] Li Y, He Y, Kawaguchi R, Zhang Y, Wang Q, Monavarfeshani A (2020). Microglia-organized scar-free spinal cord repair in neonatal mice. Nature.

[73] Babetto E, Wong KM, Beirowski B (2020). A glycolytic shift in Schwann cells supports injured axons. Nat Neurosci.

[74] Walden E, Li S (2022). Metabolic reprogramming of glial cells as a new target for central nervous system axon regeneration. Neural Regen Res.

[75] Ewan EE, Avraham O, Carlin D, Gonçalves TM, Zhao G, Cavalli V (2021). Ascending dorsal column sensory neurons respond to spinal cord injury and downregulate genes related to lipid metabolism. Sci Rep.

[76] Zhao XF, Huffman LD, Hafner H, Athaiya M, Finneran MC, Kalinski AL (2022). The injured sciatic nerve atlas (iSNAT), insights into the cellular and molecular basis of neural tissue degeneration and regeneration. Elife.

[77] van der Vlist M, Raoof R, Willemen HLDM, Prado J, Versteeg S, Gil CM (2021). Macrophages transfer mitochondria to sensory neurons to resolve inflammatory pain. Neuron.

[78] Li F, Sami A, Noristani HN, Slattery K, Qiu J, Groves T (2020). Glial metabolic rewiring promotes axon regeneration and functional recovery in the central nervous system. Cell Metab.

[79] Han Q, Xie Y, Ordaz JD, Huh AJ, Huang N, Wu W (2020). Restoring cellular energetics promotes axonal regeneration and functional recovery after spinal cord injury. Cell Metab.

[80] Jha MK, Passero JV, Rawat A, Ament XH, Yang F, Vidensky S (2021). Macrophage monocarboxylate transporter 1 promotes peripheral nerve regeneration after injury in mice. J Clin Invest.

[81] Rivlin M, Miller A, Tulipan J, Beredjiklian PD, Wang ML, Fertala J (2017). Patterns of production of collagen-rich deposits in peripheral nerves in response to injury: A pilot study in a rabbit model. Brain Behav.

[82] Fertala J, Rivlin M, Wang ML, Beredjiklian PK, Steplewski A, Fertala A (2020). Collagen-rich deposit formation in the sciatic nerve after injury and surgical repair: A study of collagen-producing cells in a rabbit model. Brain Behav.

[83] Kawasaki K, Ushioda R, Ito S, Ikeda K, Masago Y, Nagata K (2015). Deletion of the collagen-specific molecular chaperone Hsp47 causes endoplasmic reticulum stress-mediated apoptosis of hepatic stellate cells. J Biol Chem.

[84] Obata Y, Nishino T, Kuhibiki T, Tomoshige R, Xia Z, Miyazaki M (2012). HSP47 siRNA conjugated with cationized gelatin microspheres suppresses peritoneal fibrosis in mice. Acta Biomater.

[85] Sato Y, Murase K, Kato J, Kobune M, Sato T, Kawano Y (2008). Resolution of liver cirrhosis using vitamin A-coupled liposomes to deliver siRNA against a collagen-specific chaperone. Nat Biotechnol.

[86] Cattin AL, Burden JJ, Van Emmenis L, Mackenzie FE, Hoving JJA, Garcia Calavia N (2015). Macrophage-induced blood vessels guide schwann cell-mediated regeneration of peripheral nerves. Cell.

[87] Lewis GM, Kucenas S (2014). Perineurial glia are essential for motor axon regrowth following nerve injury. J Neurosci.

[88] Villegas MR, Baeza A, Usategui A, Ortiz-Romero PL, Pablos JL, Vallet-Regí M (2018). Collagenase nanocapsules: An approach to fibrosis treatment. Acta Biomater.

[89] Liao Y, Smyth GK, Shi W (2014). FeatureCounts: An efficient general purpose program for assigning sequence reads to genomic features. Bioinformatics.

[90] Love MI, Huber W, Anders S (2014). Moderated estimation of fold change and dispersion for RNA-seq data with DESeq2. Genome Biol.

[91] Subramanian A, Tamayo P, Mootha VK, Mukherjee S, Ebert BL, Gillette MA (2005). Gene set enrichment analysis: A knowledge-based approach for interpreting genome-wide expression profiles. Proc Natl Acad Sci U S A.

[92] Krämer A, Green J, Pollard J, Tugendreich S (2014). Causal analysis approaches in ingenuity pathway analysis. Bioinformatics.

