# Supplementary material for: Next-generation RNA sequencing elucidates transcriptomic signatures of pathophysiologic nerve regeneration
Source: Sci Rep. 2023 May 31;13:8856. doi: 10.1038/s41598-023-35606-6 (PMC10232541; doi:10.1038/s41598-023-35606-6)
Supplement: Supplementary file 1 — Supplementary Information. [file 41598_2023_35606_MOESM1_ESM.pdf]

Supplementary Material for
"Next-generation RNA-sequencing elucidates transcriptomic signatures of pathophysiologic nerve regeneration"

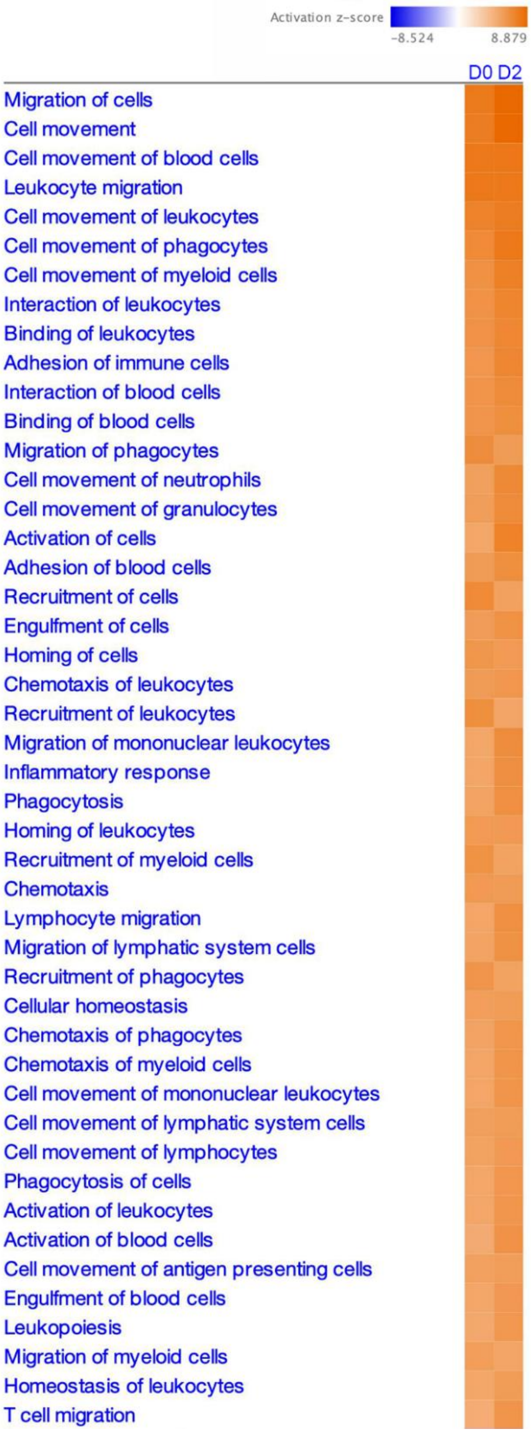

Supplementary Figure S1. Differentially expressed genes of Sham vs. Control subject to IPA Diseases and Functions Analysis (QIAGEN Inc., https://digitalinsights.qiagen.com/IPA) for Day 0 (6 hours) and Day 2 after injury. Pathways identified are overwhelmingly associated with the inflammatory response.

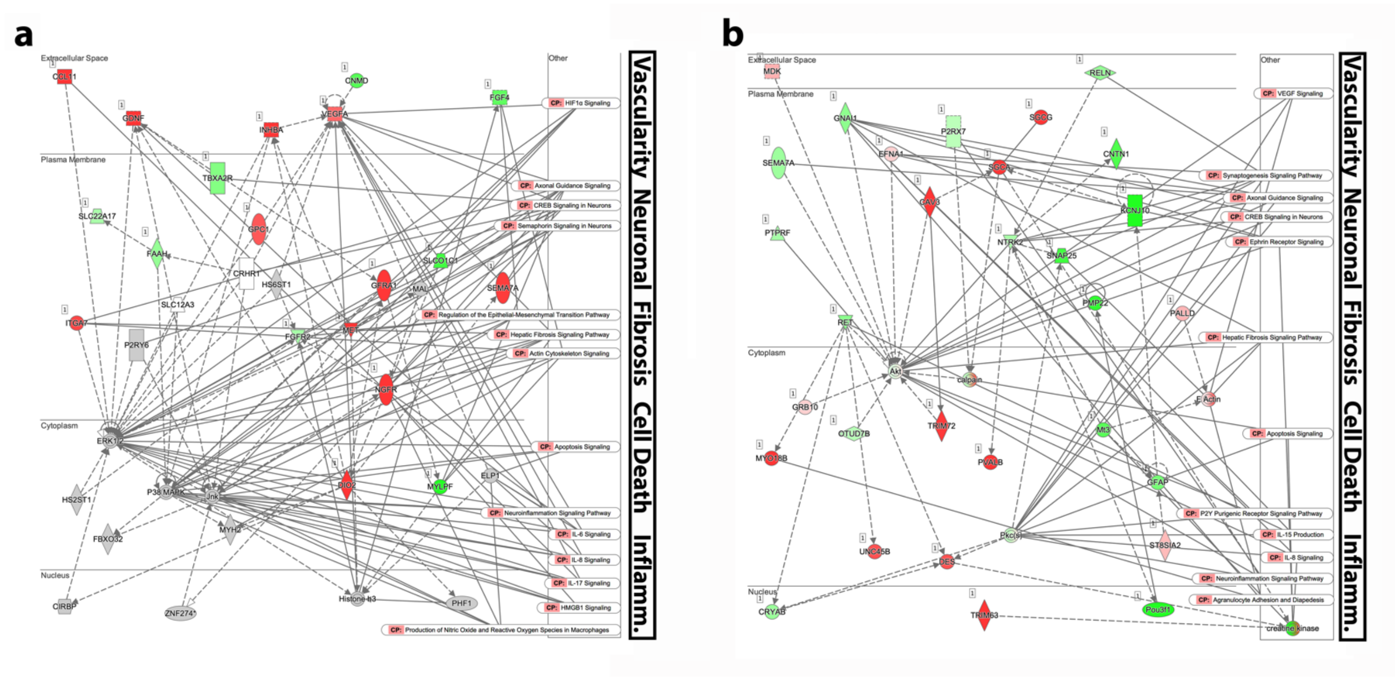

Supplementary Figure S2. Significant genes specific to the rupture injury state for (a) day 0 vs. day 2 and (b) day 2 vs. day 7, subjected to Ingenuity network analysis for construction of *de novo* genetic networks of highly interconnected genes representative of significant biological function (QIAGEN Inc., https://digitalinsights.qiagen.com/IPA). Overlay with canonical pathways permits elucidation of how these networks are associated to the relevant biological functions. Red represents significant upregulation; green down regulation, while grey are genes not in the isolated dataset, but identified as highly related. Five motifs were identified to which pathways repeatedly align: Vascularity, Neuronal, Fibrosis, Cell Death, and Inflammation. (a) Critical hub genes that are inhibited in the dataset include MYLPF, FGFR2, FGF4, and SLCO1C1 while those upregulated include VEGFA, DIO2, SEMA7A, CCL1, GDNF, and NGFR. Those that were not identified as significantly unique to the ruptured state, though highly connected, include P38MAPK, ERK1/2, JNK, MYH2, and Histone H3. Pathways associated with inflammation are largely proinflammatory, such as Production of Nitric Oxide and Reactive Oxygen Species in Macrophages, Il-17 Signaling, IL-8 Signaling, and HMGB1 Signaling. (b) Critical hub genes inhibited in the dataset include GNAI1, KCNJ10, SNAP25, PMP22, GFAP, POU3f1, and Mt3 while those upregulated include TRIM63, TRIM72, SGCA, SGCG, CAV3, and MYO18B. Neuronal signaling pathways are largely associated with axonal guidance and neurite outgrowth, while Inflammation remains hallmarked with pro-inflammatory signaling, such as IL-8 and IL-15 signaling, and also suggest specificity to the nerve through the Neuroinflammation signaling pathway.

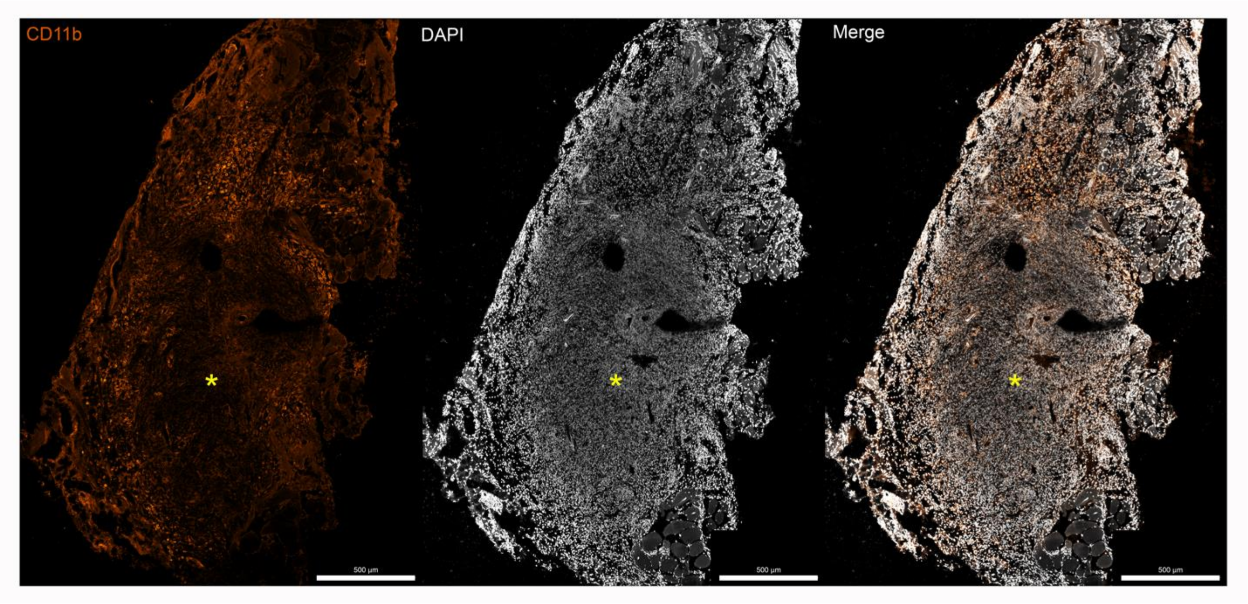

Supplementary Figure S3. Stitched confocal image (×100) of neuroma-in-continuity formation 14 days after injury at the hamstring bifurcation and zone of stretch-rupture. Core of apparent cellular death (yellow asterisk) is hallmarked by blurring of nuclear DAPI staining (white), suggestive of nuclear disintegration. Cellular death is further demonstrated by large aggregation of CD11b+ cells (orange), a pan-granulocyte marker, which have been associated with clearance of nonviable cells.

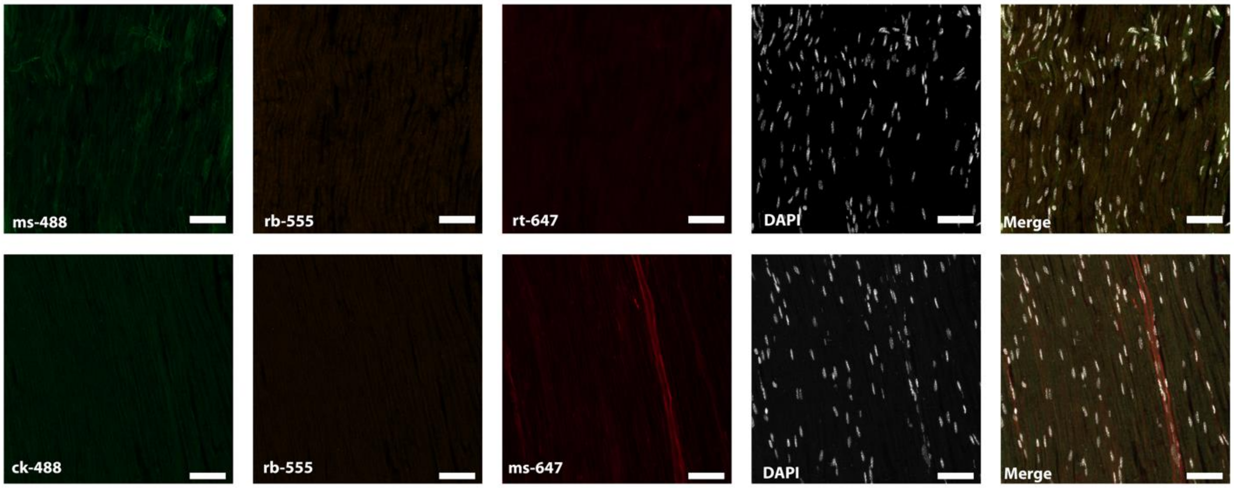

Supplementary Figure S4. Immunofluorescent staining with only secondary antibodies to assess for non-specific binding. Minimal autofluorescence or non-specific binding is observed with all secondary antibodies used. Images acquired at ×200. Scale bar, 50 μm.
